# Spontaneous Gastric Necrosis: A Rare Presentation of Invasive Mucormycosis in an Immunocompetent Adult

**DOI:** 10.1155/2020/7514051

**Published:** 2020-07-24

**Authors:** Tariq Hameed, Sudhir Kumar Jain, Faiz Manzar Ansari, Adiba Nizam, Amrita Dua

**Affiliations:** ^1^Department of Surgery, Hamdard Institute of Medical Sciences and Research, New Delhi, India; ^2^Department of Surgery, Maulana Azad Medical College, New Delhi, India

## Abstract

Spontaneous gastric perforations are usually seen in patients with untreated peptic ulcer disease. Mucormycosis, an uncommon, opportunistic, life-threatening fungal infection, rarely causes gastric perforation in immunocompetent adults. Here, we present a case of young female who was admitted to hospital for acute pain abdomen and distension with 5 days history of fever. She was operated and was found to have multiple perforations in the stomach with transmural necrosis. Despite adequate surgical excision and intravenous amphotericin B, patient succumbed to sequelae of infection.

## 1. Introduction

Stomach is a highly vascular organ, and rarely, it undergoes any necrosis. Necrosis of stomach due to invasive mucormycosis is an extremely rare entity in adults, and only few cases are reported in the literature [1]. Mucormycosis is a general term used for a spectrum of diseases caused by order mucorales, which has 53 genera out of which *Rhizopus* most commonly affects humans [2]. Invasive mucormycosis is associated with very high morbidity and mortality [1]. Invasive mucormycosis is mostly seen in immunocompromised individuals, patients with uncontrolled diabetes mellitus, on long-term steroids, individuals who have received organ transplants including bone marrow transplants, iron over load, and rarely those with history of trauma [3–9].

## 2. Case Report

A 22-year-old female presented to surgery emergency room with severe abdominal pain and progressive distension for 1 day. Pain was sudden in onset and acute in nature. She also gave a history of nonpassage of faeces and flatus for 1 day. She had fever with chills for 5 days, for which she consulted her local physician and took medicines. Fever was relieved on taking medication only to reappear again once the effect of antipyretics was over. She had no history of any major illness including diabetes. She had no history of substance abuse or steroids intake.

On physical examination, she had abdominal distension and generalised tenderness with diffuse guarding and rigidity. She had absent bowel sounds. On percussion, there was obliteration of normal liver dullness. She was hemodynamically unstable with a BP reading of 88/66 mmHg, and pulse rate was 128/minute. She was febrile, and her temperature was 103°F.

**Figure 1:**
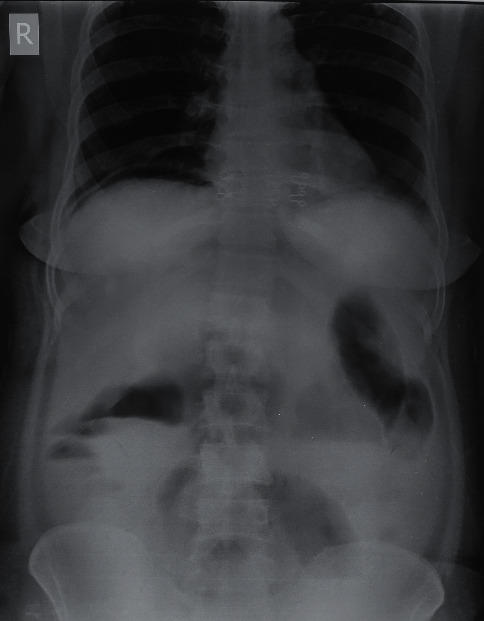
X-ray abdomen showing free gas under right dome of diaphragm.

Her laboratory reports underlined metabolic acidosis. Her TLC was 24600/*µ*l, and blood urea was 63 mg/dl. She was resuscitated with adequate hydration and inotropic support. An X-ray abdomen in erect posture revealed free gas under right dome of diaphragm (Figure 1).

**Figure 2:**
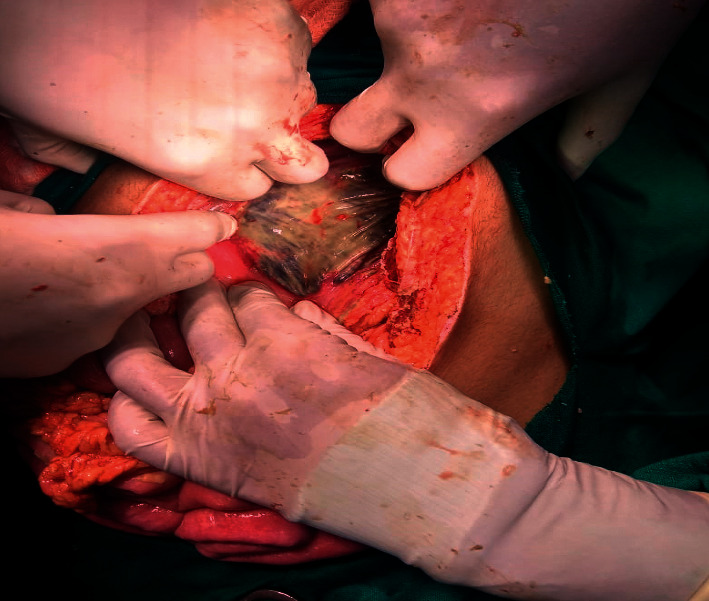
The macroscopic appearance of the transmural gastric necrosis.

In view of clinical condition and radiological finding, the patient was planned for emergency exploratory laparotomy and was shifted to operation theatre. On exploration, turbid and bile-stained fluid of approximately 1.5 litres was drained. Anterior and posterior walls of stomach were necrosed with multiple small perforations (Figure 2). Rest of the bowel was grossly normal. Total gastrectomy with feeding jejunostomy was done. Postoperative patient was shifted to ICU and was on inotropic support and broad-spectrum antibiotics. After discussion over the possible pathology and similar cases in literature, amphotericin B was started on 3rd postoperative day. But the patient deteriorated gradually and developed MODS. She could not be resuscitated and died on POD 5. Histopathological report of the patient was available on 7th day.

**Figure 3:**
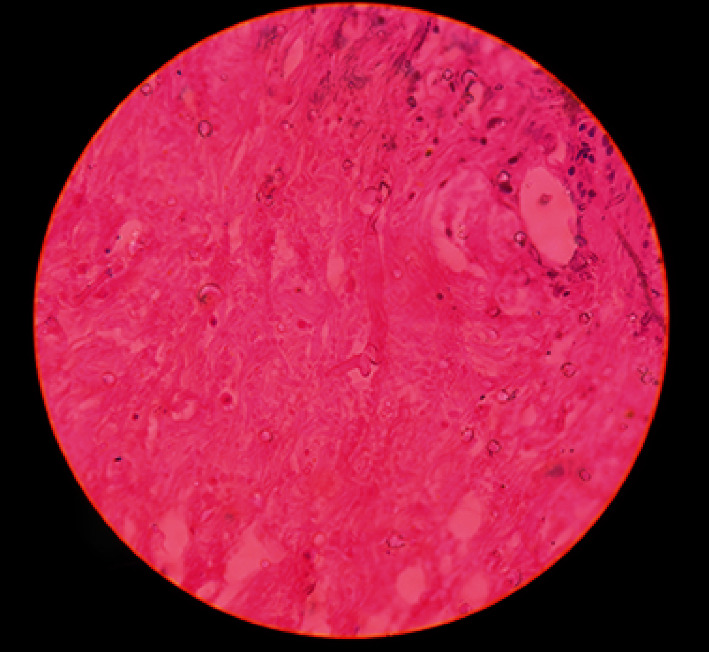
Photomicrograph of the histological appearance of mucormycosis. A gastric wall tissue section examined by light microscopy. The histology of the fungal form of mucormycosis shows large, nonseptate hyphae with 90 degree angle hyphal branching.

**Figure 4:**
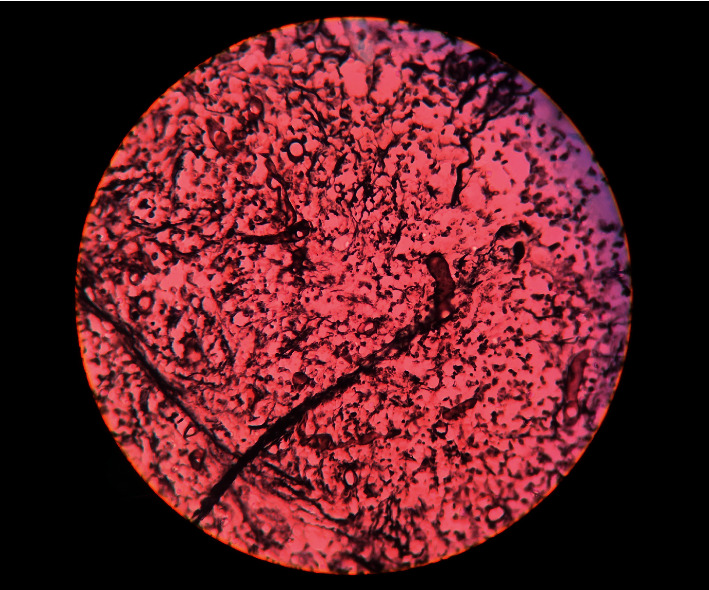
Photomicrographs showing the morphology of mucormycosis. Grocott-Gömöri methenamine silver stain (GMS) (black).

Histopathological examination showed extensive areas of ulceration with transmural acute inflammation, large mass of liquefactive necrosis with foci of entrapped broad, and aseptate ribbon-like fungal hyphae. At places, hyphae show angioinvasion (Figures 3 and 4). Histopathological findings were suggestive of mucormycosis.

## 3. Discussion

Mucormycosis has been reported to affect almost all the organs, but the most common type of presentation is rhino-orbito-cerebral (ROCM) mucormycosis [10, 11]. It is associated with diabetes mellitus, usually uncontrolled, and studies from India and USA showed that 88% and 83% patients had diabetes mellitus, respectively [12, 13]. Mucormycosis involving gastrointestinal tract is difficult to diagnose in early stages, and it usually presents with some form of complications. Gastrointestinal mucormycosis is seen classically in immunodeficient conditions: solid organ transplants, haematological malignancies, and neutropenic situations [14]. Diabetes mellitus, chronic alcoholism, poor nutrition, and peritoneal dialysis are among the major factors for gastrointestinal mucormycosis [14]. Kaur et al. reviewed 176 immunocompetent cases presenting with gastrointestinal mucormycosis. More than 50% of cases were from Asia, with equal distribution in adult and pediatric population [14]. Among all sites of gastrointestinal mucormycosis, the stomach is most common site and accounts for more than 50% of cases followed by colon and ileum [15]. Gastrointestinal mucormycosis though uncommon has very high mortality [16]. The stomach is usually involved by ingestion of infected sputum, intake of naturistic medicines, spores on tongue depressors, or in patients who already had gastric ulcers [15, 17]. Clinical presentation is nonspecific and has a spectrum of presentation ranging from pain, diarrhoea, fever, gastrointestinal bleeding, necrosis, perforation, and as necrotising enterocolitis in premature newborns [18, 19]. Colonic mucormycosis has also presented as massive lower gastrointestinal bleeding [20].

Diagnosis of mucormycosis can be established either by growth on culture or histopathological examination of tissues involved. Mucormycosis is an aggressive disease with high mortality; therefore, a combined medical and surgical approach is best suited in most of the cases. Surgical options include debridement and resection of involved organ or part of it depending on the site of involvement. Antifungal amphotericin B is the most effective and commonly used drug [14].

## 4. Conclusion

Gastrointestinal mucormycosis has very high mortality rate. Gastric perforation due to invasive mucormycosis in an immunocompetent patient is a rare presentation. Awareness of presenting symptoms, high index of suspicion, and knowledge of risk factors along with early diagnosis and prompt treatment can help in managing such patients. Systemic antifungal amphotericin B along with aggressive debridement of devitalised tissues and removal of all infected tissues is advised and has better prognosis.
